# The industrially important genus *Kaempferia*: An ethnopharmacological review

**DOI:** 10.3389/fphar.2023.1099523

**Published:** 2023-02-27

**Authors:** Arpit Singh, Nitesh Singh, Sanchita Singh, Ravi Prakash Srivastava, Lav Singh, Praveen C. Verma, Hari P. Devkota, Laiq ur Rahman, Bikash Kumar Rajak, Amrita Singh, Gauri Saxena

**Affiliations:** ^1^ Department of Botany, University of Lucknow, Lucknow, Uttar Pradesh, India; ^2^ Department of Plant-Pathology, Faculty of Agriculture and Science, SGT University, Gurgaon, India; ^3^ CSIR-National Botanical Research Institute (NBRI), Lucknow, Uttar Pradesh, India; ^4^ PG Department of Botany, R.D and D.J. College, Munger University, Munger, India; ^5^ Central Academy for State Forest Services, Burnihat, Assam, India; ^6^ Graduate School of Pharmaceutical Sciences, Kumamoto University, Kumamoto, Japan; ^7^ Pharmacy Program, Gandaki University, Pokhara, Nepal; ^8^ CSIR-Central Institute of Medicinal and Aromatic Plants (CIMAP), Lucknow, Uttar Pradesh, India; ^9^ Department of Bioinformatics, Central University of South Bihar, Gaya, India; ^10^ Department of Botany, Sri Venkateswara College, University of Delhi, Delhi, India

**Keywords:** phytochemistry, phytochemicals, ethnopharmacology, ethnobotany, Essential oil, Kaempferia

## Abstract

*Kaempferia*, a genus of the family Zingiberaceae, is widely distributed with more than 50 species which are mostly found throughout Southeast Asia. These plants have important ethnobotanical significance as many species are used in Ayurvedic and other traditional medicine preparations. This genus has received a lot of scholarly attention recently as a result of the numerous health advantages it possesses. In this review, we have compiled the scientific information regarding the relevance, distribution, industrial applications, phytochemistry, ethnopharmacology, tissue culture and conservation initiative of the *Kaempferia* genus along with the commercial realities and limitations of the research as well as missing industrial linkages followed by an exploration of some of the likely future promising clinical potential. The current review provides a richer and deeper understanding of *Kaempferia*, which can be applied in areas like phytopharmacology, molecular research, and industrial biology. The knowledge from this study can be further implemented for the establishment of new conservation strategies.

## 1 Introduction

The chronic diseases linked to lifestyle are rising alarmingly as the world’s population ages. Globally, the diet of humans is the most significant modifiable factor to control these chronic illnesses. It is established that traditional healthy eating practices and a therapeutic plant-based diet lower the risk of these diseases and increases immunity. Due to this, traditional medicinal botanical drugs especially those used as food in traditional practices are preferred and are in demand (Vandebroek and Balick, 2012; Singh et al., 2021; Hashiguchi et al., 2022; ; Thakur et al., 2022). There are more than 25,791 plant species that have medicinal value, of which 5,411 due to overexploitation are included in the Red List of Threatened Species maintained by the International Union for the Conservation of Nature (IUCN). According to a recent report by Antonelli et al. (2020), approximately 13% are classified as threatened.

Various herbal remedies are being formulated based on traditional knowledge. To prepare for upcoming pandemics, such as most recent COVID-19 pandemic since early 2020, researchers have started to explore the possibility of developing new therapies based on medicinal plants and their active components (Adhikari et al., 2021). It was observed that the use of anti-inflammatory botanical drugs as components of anti-inflammatory meals can lower the worsening of COVID-19 symptoms brought on by long-term illnesses including diabetes and obesity (Ando et al., 2021). Apart from well-established medicinal plant species, certain other botanical drugs that have been used exclusively by regional ethnic groups also play an important role in folk medicine. Due to an increase in human mobility globally, urban communities have begun to recognize these less-known species (Hashiguchi et al., 2022; Singh N. et al., 2022).

Zingiberaceae is one such family which consists of several medicinal plants known for their ethnomedicinal value not only in India but other parts of the world. They have been used as traditional medicine and as a part of cultural heritage. The most common members include *Zingiber*, *Curcuma*, *Alpinia*, *Kaempferia*, etc (Devkota et al., 2021).

*Kaempferia* genus consists of about 62 species of which 52 names are now accepted on the POWO, (2022). Engelbert Kaempfer (1,651–1,716), a German explorer, naturalist, and writer, is honoured by this generic name (Kumar et al., 2013). Several species of *Kaempferia* are widely used throughout the world as flavorants, spices and for herbal treatments. Chemical studies on *Kaempferia* plants revealed that these species could serve as a potential source of naturally derived medications with therapeutic applications (Pham et al., 2021). It is native to tropical and subtropical Asia, where it serves as a breeding habitat for many different species throughout the monsoons in Asia (Larsen and Larsen, 2006; POWO, 2022). The plants are rich in essential oils and oleoresins. The rhizomes and fruits are generally aromatic, astringent, stimulating and also consumed as food because of starch (Kumar et al., 2013). *Kaempferia galanga* L. (KG), commonly known as cekor and documented and introduced into Europe during the 17th century, is still included as an underutilized botanical drug despite its pharmacological properties. *Kaempferia parviflora* Wall. Ex Baker (KP) is the most scientifically studied plant species, and has gained attention in the past 2 decades as a revitalizer followed by *Kaempferia rotunda* L. (KR) and *Kaempferia angustifolia* Roscoe (KA) (Funk, 2013; Elshamy et al., 2019; Hashiguchi et al., 2022).

Leaves and rhiome of KG have anti-inflammatory, analgesic, nematocidal, mosquito-repellent, larvicidal, antimicrobial, anti-oxidant, and anti-allergic effects (Umar et al., 2014). KP is known as black ginger, Krachaidum or Thai ginseng, and its rhizome and leaves have antiallergenic, antimutagenic, anticholinesterase, anti-peptic ulcer and cardioprotective activities (Rujjanawate et al., 2005; Tewtrakul et al., 2008; Sawasdee et al., 2009; Azuma et al., 2011; Malakul et al., 2011). Traditionally, the rhizome of KP has been utilized to promote blood flow and increase vitality in Thailand and Laos, where it is indigenous. Tropics like Sumatra, Malaysia, Thailand and Borneo Island are known for having large populations of this species. It is commonly considered by the Hmong hill people to lower perceived effort, increase physical labour capacity, and allow them to journey for longer periods of time (Wuttidharmmavej, 2002). The rhizome of KR is used to treat fever and indigestion and speed up wound healing (Lim, 2016). Several traditional applications of the rhizomes have been documented, and they are often used in cookery as flavours and spices (Elshamy et al., 2020). It is endangered and is one of the 100 medicinal plants on the Red List that must be conserved in Southern India. It is commercially significant and is overexploited to the point where propagation material (rhizomes), also the consumable part, is in shortage (Ravikumar et al., 2000; Preetha et al., 2016). KR is a widely spread decorative plant with silver-patterned leaves and a purple blossom that may be found from India to Indonesia (Lim, 2016). The review article aims to provide an overview of the geographical distribution, phytochemistry, pharmacology, conservation, biotechnological interventions and traditional uses of *Kaempferia* species. It also highlights the latest information on the biological activities of extracts of *Kaempferia*.

## 2 Global distribution of *Kaempferia*

**FIGURE 1:**
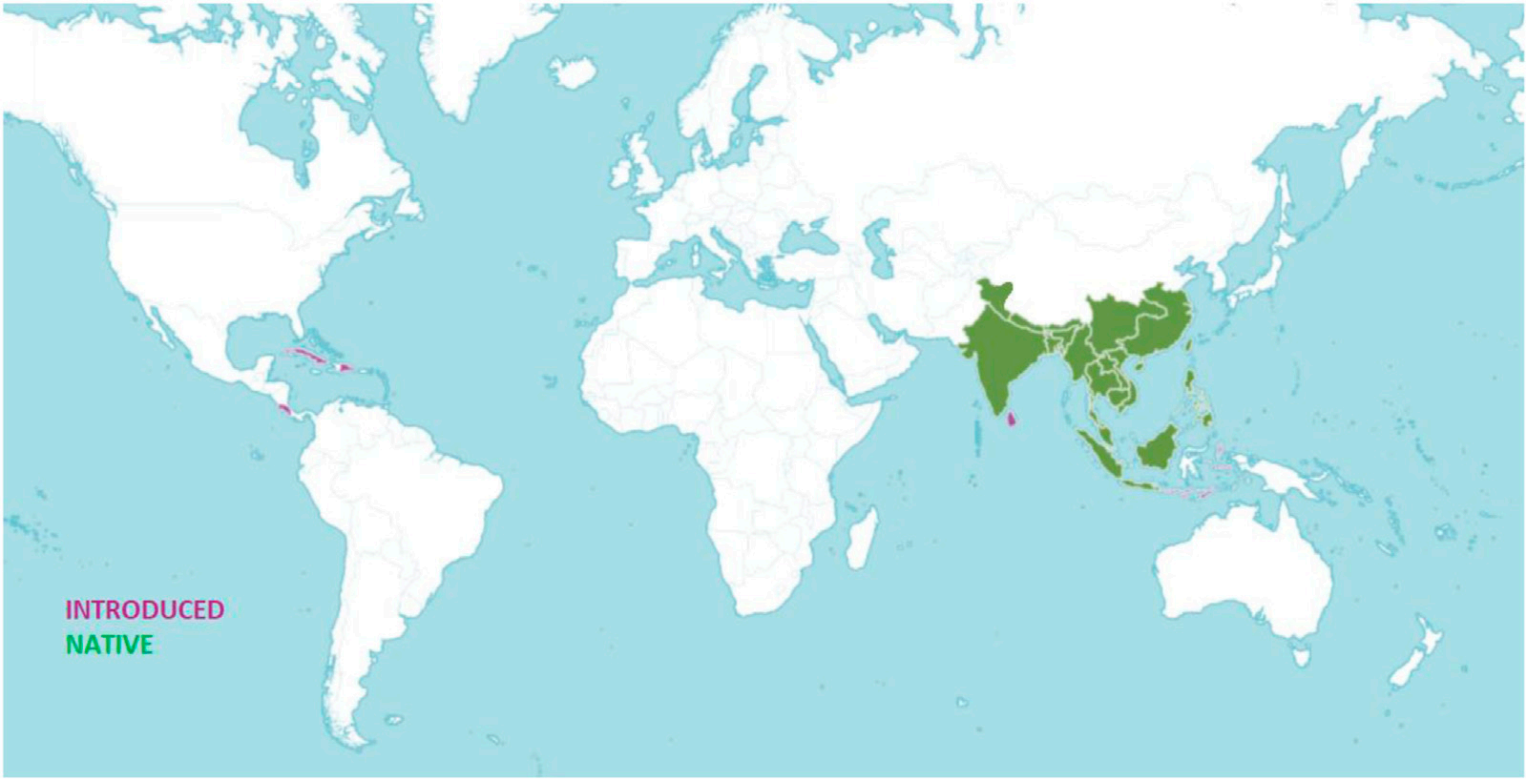
Worldwide distribution of *Kaempferia* (Source: https://powo.science.kew.org/, accessed on 15 November 2022).

Zingiberaceae is divided into 53 genera including more than 1,300 species in the world. India is one of the most varied and fertile locations for Zingiberaceae with over 200 species in 20 genera (Sabu, 2006). The *Kaempferia* genus has around 52 accepted species and is mostly found in South Asian countries like Thailand, Malaysia, Myanmar, Indonesia, the Philippines, Laos, Cambodia, and Vietnam, as well as in East Asia, specifically in China, India, and Bangladesh (Techaprasan et al.,2010; Osathanunkul et al., 2017). Of the 15 *Kaempferia* species in Thailand (Sirirugsa, 1991) twelve were discovered between 2008 and 2013 by C. Picheansoonthon and his team (Insisiengmay et al., 2019). Jenjittikul and Larsen (2000) have added the *Kaempferia* species, *Kaempferia candida* Wall. To the records of Thailand (2000). *Kaempferia grandifolia* Saensouk and Jenjitt. Is represented at Phu Phan National Park in North-East Thailand (Saensouk and Jenjittikul, 2001). Recently two new species have been discovered in Thailand namely *Kaempferia maculifolia* Boonma and Saensouk and *Kaempferia takensis* Boonma and Saensouk (Boonma et al., 2020). In India, *Kaempferia* flourishes in the northeastern states of Nagaland, Manipur, and Assam and southern states like Tamil Nadu, Kerala and Odisha in the east. Figure 1 shows the geographical distribution of the genus worldwide.

## 3 Biological aspects of *Kaempferia*

The genus includes both perennial and annual rhizomatous species. The rhizome is divided into many tubers (Kumar et al., 2013). They feature modest, typically violet or white blooms. They have spherical to fusiform tubers at the top of their typically short rhizomes, which include several fibrous roots in a fascicle. One to few leaves that are either upright or exposed to the soil (Kuehny et al., 2002; Picheansoonthon and Koonterm, 2008). Since there is a close resemblance in vegetative parts within *Kaempferia* species and other genera belonging to Zingiberaceae such as *Scaphochlamys*, *Boesenbergia*, *Caulo kaempferia*, and *Curcuma*, taxonomic identification of *Kaempferia* is challenging without the floral components (Holttum, 1950; Techaprasan et al., 2010).

KG is a geophilous botanical drug that grows all year long and has fragrant rhizomes and white tubers at the tips of its fibrous roots. It might or might not have a stem with tiny leaves. The flowers are arranged spirally and develop in the axil of bracts (Sivarajan and Balachandran, 1994).

**FIGURE 2:**
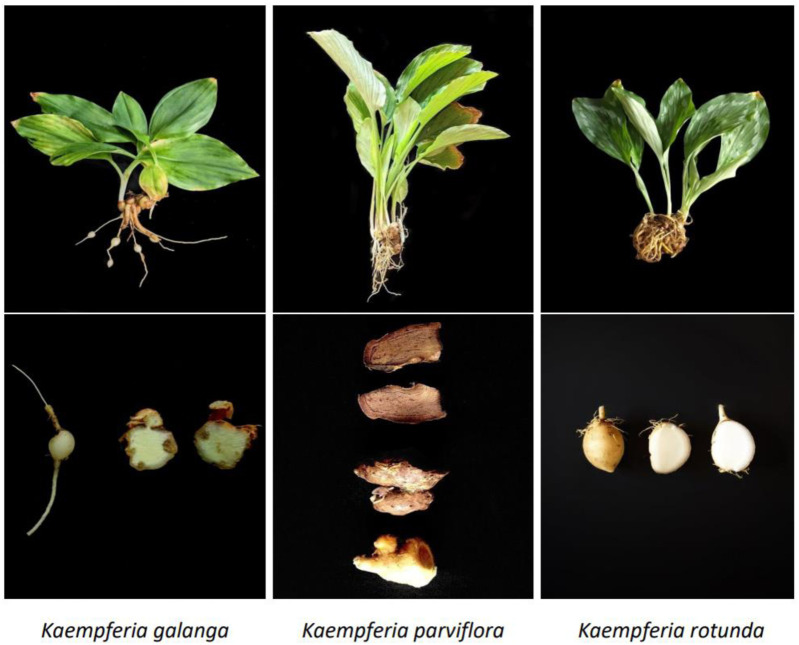
Plants and rhizome of different *Kaempferia* spp.

According to Phokham et al. (2013), the genus *Kaempferia* can be split into the KG and KR groups depending on the rate at which inflorescences develop. The KG species primarily blooms in August and September, whereas KR does so from late March to early May. KP is a perennial botanical drug that grows to a height of 90 cm and has dark purple to black rhizomes (Sae-Wong et al., 2011). Figure 2 shows different *Kaempferia* species.

## 4 Traditional uses of *Kaempferia*

As an antidote for snake venom, plants of the genus *Kaempferia* have a long history of usage in the treatment of a variety of human diseases, such as vata-related disorders like cold and cough, fever, headache, skin problems, and rheumatic conditions. The rhizomes are also highly aromatic and have been widely utilized as spices, and flavourings for food, cosmetics, and fragrance products (Kumar, 2020). In Thai traditional medicine, they are used to treat oedema, stomach ulcers, leucorrhoea, fever, and wound healing (Suksri et al., 2005; Muhammad et al., 2011; Tangjitman et al., 2015). The leaves are used to treat fever, swellings, rheumatism, and pharyngodynia (Warrier, 1993; Wutythamawech, 1997).

The most prevalent *Kaempferia* species, KG, has been significantly used as traditional medicine in many Asian countries. This component is used in over 59 Ayurvedic formulations in India to treat conditions like asthma, malaria, skin diseases, bronchitis, and wounds (Ali et al., 2018). KG is a well-known remedy for *Vata* and *Kapha* diseases and is especially helpful for respiratory conditions like cough, bronchitis, and asthma. It is used to treat splenic illnesses, wounds, and skin disorders. Various Ayurvedic preparations, such as *Valiya Rasnadi Kashayam*, *Asana Eladi Tailam*, *Dasamularistam*, *Kaccoradi Churna*, *etc.* Use the rhizome (Prabhu Kumar et al., 2010). KG is one of the 12 components of the Thai traditional remedy *Prasachandaeng*, which is used to treat respiratory and cardiovascular issues (Prommee et al., 2021; Srivastava et al., 2021). The medicine *Karcura*, which is made using KG and used to treat joint pain, asthma, hiccups, and hunger is debated as it is sometimes prepared from the *Curcuma zedoaria* (Christm.) Roscoe (Sivarajan and Balachandran, 1994). KP rhizome is used in traditional Thai medicine in the country’s north and the northeast region as an anti-cancer, anti-plasmodial, anti-allergic, and anti-gastric ulcer remedy (Mekjaruskul et al., 2012; Saokaew et al., 2017). Historically, the native people of northeast Thailand have revered these rhizomes as medicinal plants. Gout, abscesses, colic, as well as gastric and duodenal ulcers, can all be treated with them (Sae-Wong et al., 2011). Formulations for KR in Ayurveda include *Aokriam, Cyavanapram, Kalyanakaghritham, Baladhtrydi Tailam,* etc (Sivarajan and Balachandran, 1994). KR-derived hallakam is applied as an ointment on cuts and bruises to prevent them from getting worse (Warrier, 1993). Table 1 shows different ethnopharmacological uses of KG*,* KP and KR*.* Many different cuisines have used KG’s rhizome as a flavouring spice (Yao et al., 2018). The rhizomes of KG have anti-inflammatory, expectorant, diuretic, anabolic, antipyretic, anti-tussive, and carminative properties (Elshamy et al., 2019). The rhizome of KG is used to deter insects since it possesses anti-malarial, insecticidal, and nematocidal activities (Ahn et al., 2008; Choochote et al., 2007). The plant KR’s rhizome is used to treat fever and gastrointestinal ailments as well as to hasten the healing of wounds (Lim, 2016). The rhizomes of KR are thought to have antioxidant properties as well as antibacterial efficacy against harmful microorganisms like pathogenic *Escherichia coli*, *Bacillus cereus*, and *Staphylococcus aureus*. When applied topically to a fish fillet, the essential oil from the rhizome reduces the growth of microorganisms, the breakdown of proteins, and the oxidation of lipids (Diastuti et al., 2020).

**TABLE 1:** Traditional uses of *K. galanga, K. parviflora and K. rotunda.*

Species name	Distribution	Traditional uses	References
*K galanga*	Bangladesh, China, India, Taiwan, Thailand, Vietnam	• The rhizome is used in Ayurvedic preparations such as *Valiya rasnadi kashayam*, *Asana eladi tailam*, *Dasamularistam*, *Valiya Narayana tailam, Kaccoradi curna*	Pham et al., 2021; Tuan and Trong 2017; Chawengrum et al., 2018; Kumar et al., 2013
		• Treatment of menstrual stimulation and dyspepsia, skin infected with fungus *Tinea versicolor*, seizures, CNS depression and indigestion	
		• Hot leaves used as topical patches for rheumatoid arthritis, hypertension, chest and abdominal discomfort, and other conditions	
		• Used in treatment of cholera, contusions, chest problems, and headaches	
		• Indigestion is treated with essential oils extracted from rhizomes, which are used to treat constipation	
*K. parviflora*	Bangladesh, Burma, Cambodia, India, Myanmar, Thailand	• Rhizomes used to treat ailments like gout and ulcers and infections	Sirirugsa, 1991; Kobayashi et al., 2015; Pancharoen et al., 2000
		• Used in Thai folk medicine for a long time to treat leucorrhea, oral diseases, stomachaches, flatulence, digestive disorders, gastric ulcers, diuresis, and tonic, as well as an aphrodisiac agent	
		• Used to lower blood sugar levels, improve blood flow, and boost energy	
		• Taken as food supplement to help with metabolic syndrome	
*K. rotunda*	Bangladesh, China, India, Nepal, Taiwan	• Used as ornamental plant	Jagadish et al., 2016
		• Used to alleviate stomach discomfort, menstrual irregularities, insufficient menstruation, and dysmenorrhea	
		• Topical application of rhizome used in swelling and damage therapy	
		• Rhizomic decoction used to treat abdominal discomfort	
		• The entire plant crushed with salt is used to alleviate fever	
		• Rhizome is used to promote wound healing and cosmetics	

## 5 Phytochemistry

Terpenoids, flavonoids, phenolics, and essential oils possessing biological properties have been reported to date from the plants of the genus *Kaempferia*. Diterpenoids, notably isopimarane derivatives, were the most commonly reported compounds from this genus (Chawengrum et al., 2018). It was reported that the freeze-dried ethanolic extract of KG’s rhizome contained significant levels of the active substances ethyl-p-methoxycinnamate and ethyl-cinnamate (Umar et al., 2014; Adianingsih et al., 2021; Nonglang et al., 2022). Terpene, diterpene, esters, flavanones, polysaccharides, polythiourea derivatives, phenolic acids, glycosides, phenolic diarylheptanoids, Kaempferol, cystargamide B, 3-caren-5-one, xylose, and ethyl p-methoxycinnamate are just a few of the 49 phytochemicals that have been found and reported from the KG (Kumar, 2020). Table 2 lists these bioactive substances and table 3 shows their chemical structures.

**TABLE 2:** Some important chemical components isolated from *Kaempferia* spp.

Class of compound	Plant species	Compounds	References
Diterpenoids	*K. pulchra*	Kaempulchraols A-H	Win et al. (2015)
	*K. galanga*	Kaemgalangol A	Kumar (2020)
	*K. galanga*	Kaemgalangols B-D	Tungcharoen et al. (2020)
	*K. marginata*	1α-Hydroxy-14α-methoxyisopimara- 8 9),15-diene	Chokchaisiri et al. (2019)
		1α,14α-Dihydroxyisopimara-8 9),15- diene	Muderawan et al. (2022)
		Kaemgalangols E-F Marginaols A-F Sandaracopimaradiene	
Phenolic glycosides	*K. parviflora*	Kaempferiaoside A	Chaipech et al. (2012)
		Kaempferiaoside B	
Phenolics	*K. galanga*	Ethyl-*p-*methoxycinnamate	Wu et al. (2016)
		Ethyl cinnamate	Yao et al. (2018)
		*p-*Methoxybenzoicacid	Umar et al. (2014)
		*p-*Hydroxybenzoic acid	Adianingsih et al. (2021)
		Vanillic acid	
		Ferulic acid	
		Hydroxycinnamic acid, Methoxycinnamic acid	
Flavonoids	*K. pandurata*	Pinostrobin	Pandji et al. (1993)
		Cardamonin	
		Pinocembrin	
		Alpinetin	
	*K. pulchra*	2″,2″-Dimethylpyrano-[5″,6″:8,7]- flavone	Chawengrum et al. (2018)
	*K. elegans*		
	*K. parviflora*	5,7-Dimethoxyflavone	Nakao et al. (2011)
		4′,5,7-Trimethoxyflavone	Kobayashi et al. (2015)
		3′,4′,5,7-Tetramethoxyflavone	Sutthanut et al. (2007)
		3,5,7,3′,4′-Pentamethoxyflavone	Chen et al. (2018)
		5–5′-Hydroxy-7-methoxyflavone	
		5,3′-Dihydroxy-3,7,4′-trimethoxyflavone	
	*K. galanga*	Kaempferol	Umar et al. (2014)
		Kaempferide	
Steroids	*K. marginata*	*β*-Sitosterol	Kaewkroek et al. (2013)
		*β*-Sitosterol-*β*-D-glucoside	Tang et al. (2011)
		Stigmasterol	
		(24*S*)-Methyl-lanosta- 9 (11), 25-dien-3*β*-ol	
Essential oil components	*K. galanga*	δ-Selinene	Fan et al. (2005)
		n-Pentadecane	Raina and Abraham (2016)
		Eucalyptol	Yang et al. (2018)
		Borneol	Bhuiyan et al. (2008)
		Caryophyllene	
		Cubenol	
		2-Propenoic acid, 3-(4-methoxyphenyl),- ethyl ester	
		4-Cyclooctene-1-methanol Limonene	
	*K. parviflora*	*α*-Copaene	Pripdeevech et al. (2012)
		Dauca-5,8-diene	
		Camphene	
		*β*-Pinene	
		Linalool	
	*K. rotunda*	Benzyl benzoate	Woerdenbag et al. (2004)
	*K. angustifolia*	Bornyl formate	
		Camphor	

### 5.1 Monoterpenoids/diterpenoids

The rhizome of *Kaempferia pulchra* Ridl. from Myanmar yielded eight unique diterpenoids, kaempulchraols A–H, as well as five previously discovered ones (Win et al., 2015). Along with 20 other known chemicals from KG, the three novel polyoxygenated isopimarane diterpenoids known as kaemgalangols B-D were discovered (Elshamy et al., 2020). Twenty-six terpenoids, including monoterpenoids, diterpenoids, and sesquiterpenoids, have recently been identified. These were mostly isopimarane diterpenoids with two double bonds of type 15(16), 8(9), or 8(14) (Wang et al., 2021). The hexane portion of the KG ethanol extract was used to isolate compounds such as - sandaracopimaradiene, sandaracopimaradiene-1,9-diol, sandaracopimaradiene-7,9-diol, 6-6-acetoxysandaracopimaradiene-1,9-diol, 6β-acetoxysandaracopimaradiene-9α-ol (Tungcharoen et al., 2020).

Along with the previously known chemicals, marginaol A-F, two other diterpenoids, kaemgalangol E-F, have been discovered from the rhizome’s dichloromethane/methanol extract in KG (Elshamy et al., 2021). From the oils of KG rhizome obtained by maceration method, several long-chain alcohols, carboxylic acids, diterpene sandaracopimaradiene, alkaloid 2-imino-3-(3- nitrophenyl)-1,3-thiazolidin-4-one, and steroid ergosterol were also isolated. These also included 9E,12E-octadeca-9,12-dien-1-ol (Muderawan et al., 2022).

### 5.2 Phenolic and flavonoids

Flavonoids and other phenolic compounds are one of the most prevalent compounds in *Kaempferia*, specifically polymethoxy flavonoids (PMF). The phenolics (benzoyl and cinnamoyl) and flavonoids in the KG rhizome were isolated using chromatographic methods and identified by using different spectroscopic methods (Wahyuni et al., 2021). Along with 24 recognized compounds, two novel phenolic glycosides named kaempferiaosides A and B were extracted from the rhizomes of KP (Chaipech et al., 2012). *Kaempferia* was found to have 16 phenolic components, according to Wang et al. (2021). Among these are derivatives of hydroxycinnamic acids and hydroxybenzoic acids (Wu et al., 2016; Yao et al., 2018). Pinostrobin, cardamonin, alpinetin, and pinocembrin were reported from *Kaempferia pandurata* Roxb (Pandji et al., 1993), and *Kaempferia elegans* (Wall.) Baker and *K. pulchra* Ridl. Provided 2″,2″-dimethylpyrano-[5″,6″:8,7]-flavone (Chawengrum et al., 2018).

### 5.3 Steroids and triterpenoids

The rhizomes of *Kaempferia marginata* Carey ex Roscoe contained *β*-sitosterol, *β*-sitosterol-D-glucoside, and stigmasterol (Kaewkroek et al., 2013). Additionally, KA yielded one triterpene of the lanostane type, (24S)-24-methyl-lanosta-9 (11) (49), 25-dien-3-ol (Tang et al., 2011).

### 5.4 Essential oils

Terpenes, hydrocarbons, esters, and aromatic chemicals make up most essential oils. The 19 main constituents of essential oils of KG are comprised of terpenoids and esters (Fan et al., 2005; Raina and Abraham, 2016; Yang et al., 2018). Market prices for the essential oils of KG range between 600 and 700 US dollars per kilogram, making them a profitable market both in India and abroad. Trans-ethyl cinnamate, a phenylpropanoid component was discovered to be the main compound present in the essential oils of the *Kaempferia* spp (Munda et al., 2018). Following analysis of KG’s leaf and rhizome essential oils, 108 and 81 components were identified respectively. The major components were linoleoyl chloride (21.4%), caryophyllene oxide (11.7%), cubenol (9.6%) and caryophyllene (5.6%). 2-Propenoic acid, 3-(4-methoxyphenyl),-ethyl ester (63.3%), ethyl cinnamate (6.3%), 4-cyclooctene-1-methanol (4.6%), caryophyllene oxide (4.3%), and limonene (3.2%) (Bhuiyan et al., 2008).

There were at least 20 different substances in the essential oils of dried KP rhizomes. A-copaene (11.6%), dauca-5, 8-diene (11.1%), camphene (8.7%), a-pinene (7.18%), borneol (7.0%), and linalool (6.6%) was the principal component that was isolated (Pitakpawasutthi et al., 2018). This finding was consistent with another investigation on the essential oil in KP rhizomes that found that borneol (10.2%), pinene (8.6%), camphene (7.6%), copaene (7.2%), and linalool (6.4%) were the major components (Pripdeevech et al., 2012).

**FIGURE 3:**
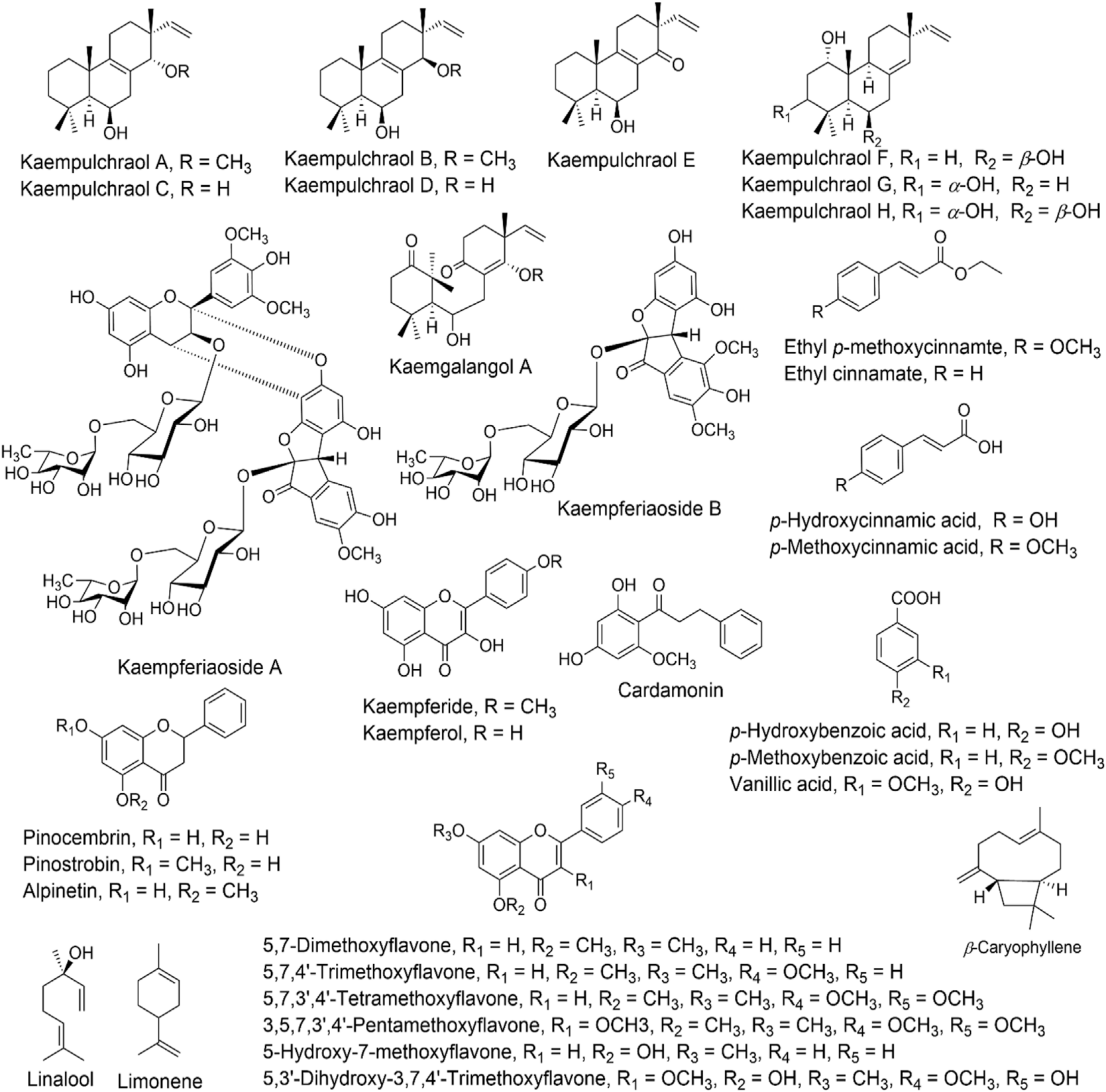
Chemical structure of the various compounds present in different parts of *Kaempferia*.

A total of 75 compounds were found when the volatile components of the main and lateral sections of two rhizomes from the plants KR and KA were analysed. N- pentadecane, benzyl benzoate, and camphene were the three most prevalent substances in the major rhizome portions of KR, while n-pentadecane, camphene, bornyl formate, and camphor were the four most common substances in KA (Woerdenbag et al., 2004). In general, it was established that altitude and location both significantly affected the distinct volatile elements both in quality and quantity. Chemical structure of the various compounds present in different parts of *Kaempferia* are provided in Figure 3.

## 6 Pharmacological and other multifarious properties

**FIGURE 4:**
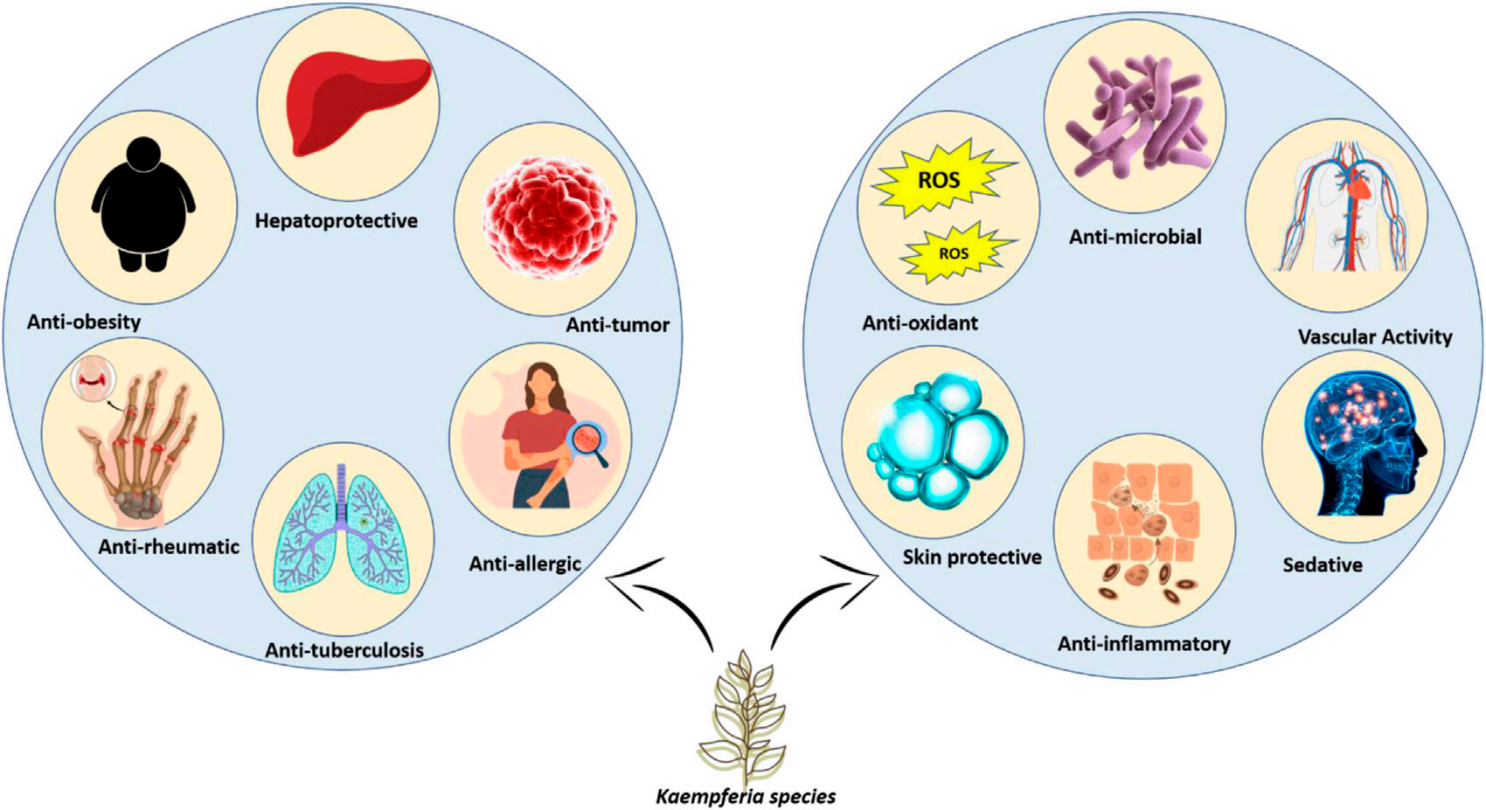
Modern pharmacological activities of *Kaempferia* species.

*Kaempferia* plants having various biological activities have received a considerable research interest in recent years. The plant extracts and natural compounds possess a wide range of bioactivities including antioxidant and anticancer properties as well as analgesic, anti-inflammatory, and anti-tuberculosis properties. Extracts from KP rhizomes (90 mg/day) have been used to treat a variety of conditions, including erectile dysfunction, hypertension, inflammation, and stomach problems (Saokaew et al., 2017). KP ethanolic extracts at a dose of 100 or 200 mg/kg/day for 8 weeks, boost energy output and fight fat by inhibiting the expression of adipogenic transcription factors and lipogenic enzymes by upregulating AMP-activated protein kinase (AMPK) in epididymal fat (Matsushita et al., 2015; Lee et al., 2018). The ethanolic extract has shown to promotes reproductive health when given at 70 mg kg^−1^ day^−1^ for 4 weeks to male rats and treats skin conditions by reducing melanogenesis and photoaging (Chaturapanich et al., 2012; Park et al., 2014; Ninomiya et al., 2016). It enhances mental wellness when given at 100 mg/kg *via* oral gavage to male sprague dawley rats and decreases the growth of cancer cells and gastrointestinal ulcers (Welbat et al., 2016; Potikanond et al., 2017; Leesombun et al., 2019). Additionally, it lessens the signs of cardiovascular diseases, sarcopenia, and inhibits the development of osteoarthritis (Kobayashi et al., 2018; Kim and Hwang, 2020). *Entamoeba histolytica* and drug-resistant *Mycobacterium* TB strains are also lysed by KG yielded ethyl *p-*methoxycinnamate (EPMC) (Lakshmanan et al., 2011). *In vitro* testing demonstrates that ethanolic extract of KG at 50 μg/mL concentration is effective against multidrug-resistant *Plasmodium falciparum* strains (Thiengsusuk et al., 2013). Researchers have identified several biological effects of this plant, including analgesic, antibacterial, antioxidant, amebicidal, anti-dengue, anti-inflammatory, anti-tuberculosis, hypo triglyceridemic, hypopigmentary, and osteolysis (Kumar, 2020). KR’s rhizome contains lectin, a potent anti-cancer drug. According to previous research, it induces cell cycle arrest in Ehrlich- Lettre ascites carcinoma and colon cancer cells through caspase-3-dependent pathways. Additionally, lectin controls the expression of genes related to apoptosis and the cell cycle (Ahmed et al., 2017; Islam et al., 2019). When given to mice intraperitoneally, an injection of KR rhizome extract in the form of silver/silver chloride nanoparticles at 6 and 12 mg/kg/day doses prevented the growth of tumours (Kabir et al., 2020). To target, the desired pharmacokinetic profile, and unfavourable side effects have all been considered in clinical research on nanoparticle drug delivery methods to maximize therapeutic effectiveness (Zhang et al., 2008). The most thorough pharmacological evaluations have been undertaken and are included below (Figure 4).

### 6.1 Anti-cancer activity

Active components of KG rhizome extract have been shown to suppress a number of cancer cells, including gastric, colon, oral, and multiple myeloma. These include cytotoxicity, apoptosis, and the inhibition of tumour cell proliferation. It might affect the HepG2 cells’ cell cycle progression and cause apoptosis (Liu et al., 2010). CL-6 cell growth was reduced by 125 and 250 μg/mL ethanolic extract by 80 and 94 per cent, respectively, with (Inhibitory Concentration) IC_50_ values of 64.2 and 49.19 μg/mL (Amuamuta et al., 2017).

Using the MTT test, the isopimarene diterpenoids compounds sandaracopimaradine-9-ol, kaempulchraol I and kaempulchraol L from the rhizome of KG showed anti-cancer activity in human HeLa (IC_50_ 75.1, 74.2, and 76.5 μM) and HSC-2 (IC_50_ 69.9, 53.3, and 58.2 μM, respectively) cancer cells (Swapana et al., 2018). In HSC-3 and Ca922 cell lines, trans-ethyl p-methoxycinnamate significantly damages the cells (Ichwan et al., 2019). Through modifying proliferation, invasion, angiogenesis, apoptosis, and inflammation in DMH-induced rat colon cancer, trans-p- methoxycinnamic acid has given (40 mg/kg b. wt.) p. o. Every day during different time periods for 30 weeks, which reversed significantly to normal from cancer (Gunasekaran et al., 2019). The KR rhizome’s lectin inhibited tumour growth *in-vivo* in Ehrlich ascites carcinoma bearing Swiss albino mice by inducing apoptosis and anticancer activity against Ehrlich ascites carcinoma cell lines (Ahmed et al., 2017).

The ethanolic extract of KG, and its bioactive components ethyl-p-methoxycinnamate (EPMC) and 5-fluorouracil (5-FU) were evaluated against CCA cell line (CL-6) using MTT assay and ICR mice model. They showed IC_50_ values of 64.2 (57.76–72.11) and 49.19 (48.16–52.29) μg/mL, respectively. 5-FU IC_50_ was 107.1 (103.53–109.64) μg/mL. Toxicity testing showed no overt harmful impact up to the maximum single oral dose of 5,000 mg/kg body weight and up to 1,000 mg/kg/day for 30 days. The extract at the maximal tolerable dose of 1,000 mg/kg body weight for 30 days showed remarkable anti-CCA efficacy in CL6-xenografted nude mice, inhibiting tumour growth (58.41%) and lung metastasis (33.3%) and prolonging survival (62 days) (Amuamuta et al., 2017).

Quercetin 3,5,7,3′,4′-pentamethyl ether (KPMF-8), a natural STAC (sirtuin-activating compound) from KP, directly interacts with SIRT1 (Sirtuin1, a NAD + -dependent deacetylase, is an essential regulator that produces multiple physiological benefits, such as the prevention of cancer and age-related diseases) and stimulates SIRT1 activity by increasing SIRT1’s binding affinity with Ac-p53 peptide, a native substrate peptide. KPMF-8 increased SIRT1-Ac-p53 peptide binding 8.2-fold, whereas resveratrol was just 1.4-fold (Zhang et al., 2021).

### 6.2 Anti-obesity activity

In a study by Akase et al. (2011), Tsumura Suzuki Obese Diabetes (TSOD) mice were given 1 to 3 percent extracts of KP for 8 weeks. The treated mice showed a suppression of all abnormalities namely, body weight gain, abnormal lipid metabolism, hyperinsulinemia, visceral fat accumulation, insulin resistance, glucose intolerance, hypertension, and peripheral neuropathy (Akase et al., 2011). Shimada et al. (2011) found that pancreatic lipase is strongly inhibited by ethyl acetate extract of KP and its component PMFs (polymethoxyflavones), which may help to prevent obesity and other metabolic diseases. Hidaka et al. (2017), used TSOD mice as an obesity model in their study and found that PMFs reduced the buildup of the subcutaneous fat layer.

### 6.3 Anti-microbial activity

*Escherichia coli, Staphylococcus aureus, Pseudomonas, Aspergillus*, and *Candida albicans* are all susceptible to the antibacterial properties of KG rhizome extract (Rao and Kaladhar, 2014). Using a disc diffusion assay with 10 μL of impregnated disc with ethanolic and methanolic extracts of KG showed suppression of different pathogenic bacteria and fungi with highest inhibition zone (21.3 ± 0.08) against *Staphylococcus aureus* (Kochuthressia et al., 2012). Similar to this, an agar well diffusion test using the ethanolic extract of KG showed considerable antifungal activity against *Malassezia* spp. With a minimum inhibitory concentration (MIC) value of 5 μg/ml (Parjo et al., 2018). Its essential oil is active against *Salmonella typhimurium* and *Staphylococcus aureus* but not against *E. coli* (Yang et al., 2018). Additionally, KG essential oils significantly acted as larvicidal agents (Panyakaew et al., 2017).

### 6.4 Anti-inflammatory activity

People have historically employed KG’s anti-inflammatory effects to relieve toothaches and stomach pain. Nitric oxide synthase (iNOS) and cyclooxygenase-2 (COX-2) mRNA expressions were used to test KG’s anti-inflammatory mechanism. The production of prostaglandin E2, a strong inflammatory mediator, was reduced by 92 per cent with an IC_50_ value of 9.2 μg/mL in *in-vitro* trials using the ethanol extract of KP. The chemical and plant extract dramatically decreased iNOS mRNA expression but not COX- 2 mRNA expression. Chloroform and hexane fractions were shown to be the most effective in *in-vivo* trials for reducing rat paw edoema (Sae-Wong et al., 2011). These and other flavonoids considerably decreased NO synthesis in lipopolysaccharide-stimulated RAW 264.7 cells, barely inhibited the production of TNF, and significantly in a dose-dependent manner decreased the expression of iNOS mRNA and protein. NF-B is activated during the inflammatory process (Sae-Wong et al., 2011). Additionally, KG rhizome diarylheptanoids reduced LPS-induced NO production in RAW 264.7 cells more effectively than indomethacin. These results support the conventional usage of KP rhizomes in the treatment of inflammatory diseases (Tewtrakul et al., 2009). It was discovered that *trans*-ethyl p-methoxycinnamate reduced inflammation *in-vitro* in both rat cotton pellet granuloma and human macrophage cell lines (U937). Granuloma development and IL-1 and TNF- production from rat granulomas decreased in both *in vivo* as well as *in-vitro* models (Umar et al., 2014). In an MTT (3-(4, 5-dimethylthiazolyl-2)-2, 5-diphenyltetrazolium bromide) assay, kaempferol induced inflammation in lipopolysaccharide-stimulated HMC-1 mast cells. IL-6, IL-8, IL-1, and TNF- secretion are considerably decreased at 40 mol/L (Zhou et al., 2015). The diarylheptanoids in LPS suppress the NO synthesis in macrophage RAW264.7 cells. Their respective IC50 values were 27.85, 46.98, 26.98, and 17.26 mM (Yao et al., 2018).

### 6.5 Anti-Tuberculosis activity

Trans-ethyl p-methoxycinnamate’s anti-TB activity against the bacterial strains H37Ra and H37Rv was evaluated using the Resazurin microtiter test. MIC values for trans-ethyl p-methoxycinnamate ranged from 0.242 to 0.485 mM, indicating significant anti-tuberculosis activity. According to the research, the substance has potent anti-tuberculosis properties. This work established the anti-tuberculosis properties of KG and its isolate trans-ethyl p-methoxycinnamate; nevertheless, more studies into the molecular mechanisms of action and clinical trials are required (Lakshmanan et al., 2011).

### 6.6 Anti-allergic activity

RBL-2H3 cells, which are generated from rat basophile leukaemia, have shown antigen-stimulated degranulation to be effectively suppressed by PMFs isolated from the KP. Strong inhibitory activities of the flavonoids five-hydroxy-3,7,4′-trimethoxyflavone and five-three- dihydroxy-3,7,4′-trimethoxyflavone were discovered. These effects were connected to the inhibition of degranulation brought on by Ca^2+^ influx and the translocation of the IgE receptor FcRI to the cell surface, respectively. The cell-bound IgE-FcRI complex becomes cross-linked as a result of an antigen, which causes FcRI to congregate. Numerous cellular processes are brought on by this FcRI clumping, including the production of chemical mediators like histamine, arachidonate metabolites, and cytokines. The most typical allergic reaction is a type I. Some of the symptoms of these types of allergies may be lessened by PMFs of KP (Kobayashi et al., 2015).

### 6.7 Sedative activity

By decreasing the activity of locomotor neurons, KG is well known for its sedative effects. Trans-ethyl p-methoxycinnamate and trans-ethyl cinnamate are among the substances extracted from a methanolic extract that have noticeable sedative effects (Huang et al., 2008). The acetone extract of KG showed sedative action in Swiss albino mice when provided at a concentration of 200 mg/kg body weight per oral (Ali et al., 2015).

### 6.8 Anti-rheumatic and anti-osteoporosis activities

Kaempferol, a flavonoid that is abundant in the KR inhibits the MAPK pathway prevented rheumatoid arthritis fibroblast-like synoviocytes (RA-FLSs) from migrating, invading, and expressing matrix metalloproteinases (MMPs), which markedly reduced the production of tumour necrosis factor (TNF-). As a result, kaempferol prevented cartilage degeneration and slowed the course of rheumatoid arthritis (rheumatic arthritis). Additionally, the flavonoids 5,7- dimethoxyflavone and 5,7,4′-trimethoxyflavone, which are found in the *KP* extract, reduced the expression of extracellular MMPs and collagen degradation in cartilage (Thao et al., 2016). Arthritis-prone mice showed a lower pain threshold and less severe osteoarthritic cartilage lesions (Kobayashi, et al., 2018).

### 6.9 Hepatoprotective activity

KP altered the cytochrome P450 enzymes in the liver. The activity of numerous CYP450 enzymes was dramatically increased by KP extract. Among the CYP1A2 enzymes, it showed the highest Vmax (15.276 0.206 nmol/min) and the lowest Ki value (0.008 0.002 g/mL). Therefore combining it with drugs or other plants should be avoided to avoid potential drug-botanical drug interactions (Mekjaruskul et al., 2012).

### 6.10 Aphrodisiac effect

To treat erectile dysfunction, many molecular targets are being investigated. One of the most often targeted proteins is Phosphodiesterase-5 (PDE-5). It was observed to be suppressed by KP rhizome extract and 7- methoxyflavone component. PDE-5 inhibition reduced ED, relaxed muscles, raised intracellular cGMP content, and improved blood flow to the corpus cavernosum (Khanh et al., 2018). According to Stein et al. (2018), healthy men’s erectile function was improved by extracts of KP rhizomes, standardized to 5 percent DMF.

The most effective PDE-5 inhibitors were 3,5,7,3′,4′-pentamethoxyflavone (IC50 = 30.41 M) and 5,7-dimethylflavone (IC50 = 10.64 M). Sildenafil, with an IC50 of 0.0068 M, served as the positive control. The traditional use of KP to enhance sexual performance is supported by this study. Additionally, 5,7-dimethoxyflavones could be utilised to create PDE5 inhibitors that are clinically efficacious (Horigome et al., 2016). In mouse testis-derived cancer cells, cAMP response element binding protein signalling was also demonstrated to increase testosterone synthesis, pointing to potential additional advantages (Temkitthawon et al., 2011). The administration of a KP extract in an aqueous solution containing 1% Tween-80 increased testosterone levels, sperm count, and sexual function in streptozotocin-induced diabetic rats (Lert-Amornpat et al., 2017).

### 6.11 Antithrombotic effect

Mice treated with a collagen and adrenaline-induced thrombotic paradigm were administered an oral ethanolic extract of KG. The mice received oral doses of 7, 14, and 28 mg extract per 20 g body weight. It has been shown that mice pre-treated with extract had 7-day survival rates and prolonged bleeding times. Therefore, it was concluded that the *KG* extract’s strongest antithrombotic potency was comparable to the positive control (aspirin) when given at a high dose (28 mg per 20 g body weight) in this investigation. It will take more thorough research to determine its effectiveness as an antithrombotic agent (Saputri and Avatara, 2018).

### 6.12 Vasodilatory activity

In a previous study it was concluded that trans-ethyl cinnamate possessed vasorelaxant properties which further supported the fact that traditionally, KG was used in the treatment of high blood pressure. A dose-dependent suppression of tonic contractions brought on by high potassium (K^+^) and phenylephrine (PE) doses is possible. Mechanistic investigations reveal that its vasorelaxant effect is linked to endothelial cell prostacyclin and NO release, as well as a decrease in Ca^2+^ influx into vascular cells. The botanical drug’s historical use as a hypertension medication can be explained by its vasorelaxant effects (Othman et al., 2002). A dichloromethane extract of KG was administered to anaesthetized rats, and it was discovered to have a vasorelaxant effect by reducing their basal mean arterial pressure (MAP). Furthermore, trans-ethyl cinnamate was isolated and fractionated using bioassay-guided fractionation and separation to identify the active component (Othman et al., 2006).

### 6.13 Skin effect and wound-healing activity

Increased collagen levels in the wound help with wound healing when KG rhizome extract is administered (Shrivastav et al., 2018). The anti-gastric ulcer action of the ethanolic extract of KP rhizomic powder in mice was not related to a reduction in stomach acid secretion but rather to the preservation of gastric mucus secretion (Rujjanawate et al., 2005). It was possible for the rhizome of KG, which contains isoamyl p-methoxycinnamate and other ingredients, to serve as an active photostabilizing agent and offer UV absorption for sunscreen products (Gonzalez et al., 2002). It can also be employed in the pharmaceutical and cosmetic industries for exterior applications because they have produced positive outcomes like improving skin moisture, reducing wrinkles and whitening the skin (Hwang and Kim 2014). The KP extract reduced triglyceride and fat accumulation in sebocytes, reducing skin infections and functioning as a natural acne treatment (Jin an Lee, 2018). Additionally, KG plant rhizomic extract has been used as compositions for personal care products (Kummee et al., 2008; Srivastava et al., 2019).

### 6.14 Anti-helminthic, anti-amoebic, mosquito repellent and larvicidal activities

The researchers found that a methanolic extract of KG containing cinnamate groups like ethyl cinnamate, ethyl p-methoxycinnamate, and p-methoxycinnamic acid has larvicidal activity against *Toxocara canis* second stage larvae, *Spodoptera littoralis* (Pandji et al., 1993) neonate larvae, and other larval stages of several other species (Kanjanapothi et al., 2004). *Anopheles barbirostris*, *Anopheles aconitus*, *Mansonia uniformis*, and *Aedes aegypti* were among the pests against which the extract and fractions of KG were tested. The extract and fractions were discovered to have larvicidal efficacy against such mosquito species as well as repellant activities (Kanjanapothi et al., 2004). The results suggest that KG may be combined with other strategies to combat mosquito-borne illnesses such as malaria, dengue fever and zika.

### 6.15 Anticholinesterase activity

Anticholinergic pharmaceuticals are a class of medicines that block the body’s naturally occurring neurotransmitter acetylcholine in the central and peripheral nervous systems. Numerous illnesses linked to the stimulation of the parasympathetic nervous system are treated using this class of drugs. 7-methoxyflavones, isolated from KP extracts demonstrated strong inhibitory effects on the acetylcholinesterase (AChE) and butyrylcholinesterase (BChE) enzymes (Sawasdee et al., 2009). AChE inhibitors, also referred to as anti-cholinesterases, increase the strength and duration of the effects of the neurotransmitter by preventing ACh from being degraded by the cholinesterase enzyme.

### 6.16 Anti-mutagenicity activity

A biological system may undergo heritable modifications as a result of mutagenicity, which is the development of irreversible changes in an organism’s DNA sequence. Antimutagenic medications can undo the effects of mutagens. Both anti-mutagenicity and glucosidase inhibitory activity were present in KP. According to studies, substance 7- methoxyflavones was a significant component of both extracts and showed antimutagenic action with an IC_50_ value of 0.40 nmol/plate (Azuma et al., 2011). Three flavanones, 5-hydroxy-7- methoxyflavanone, 7- hydroxy-5-methoxyflavanone, and 5,7-dihydroxyflavanone were extracted from dried and ground KR rhizome. Atun et al. (2013) also reported the methanol extract and flavanones from KR with antimutagenic action.

The 90-day toxicity and genotoxicity studies indicate that KP extract is safe for ingestion as a functional food or supplement. KP extract did not cause gene changes in bacteria or toxicity in male or female rats after 90 days of repeated oral administration at a high dose (249 mg/kg bw/day, corresponding to 747 mg KPFORCETM/kg). Its no-observed-adverse-effect level in rats is > 249 mg/kg bw/day (Yoshino et al., 2019).

**FIGURE 5:**
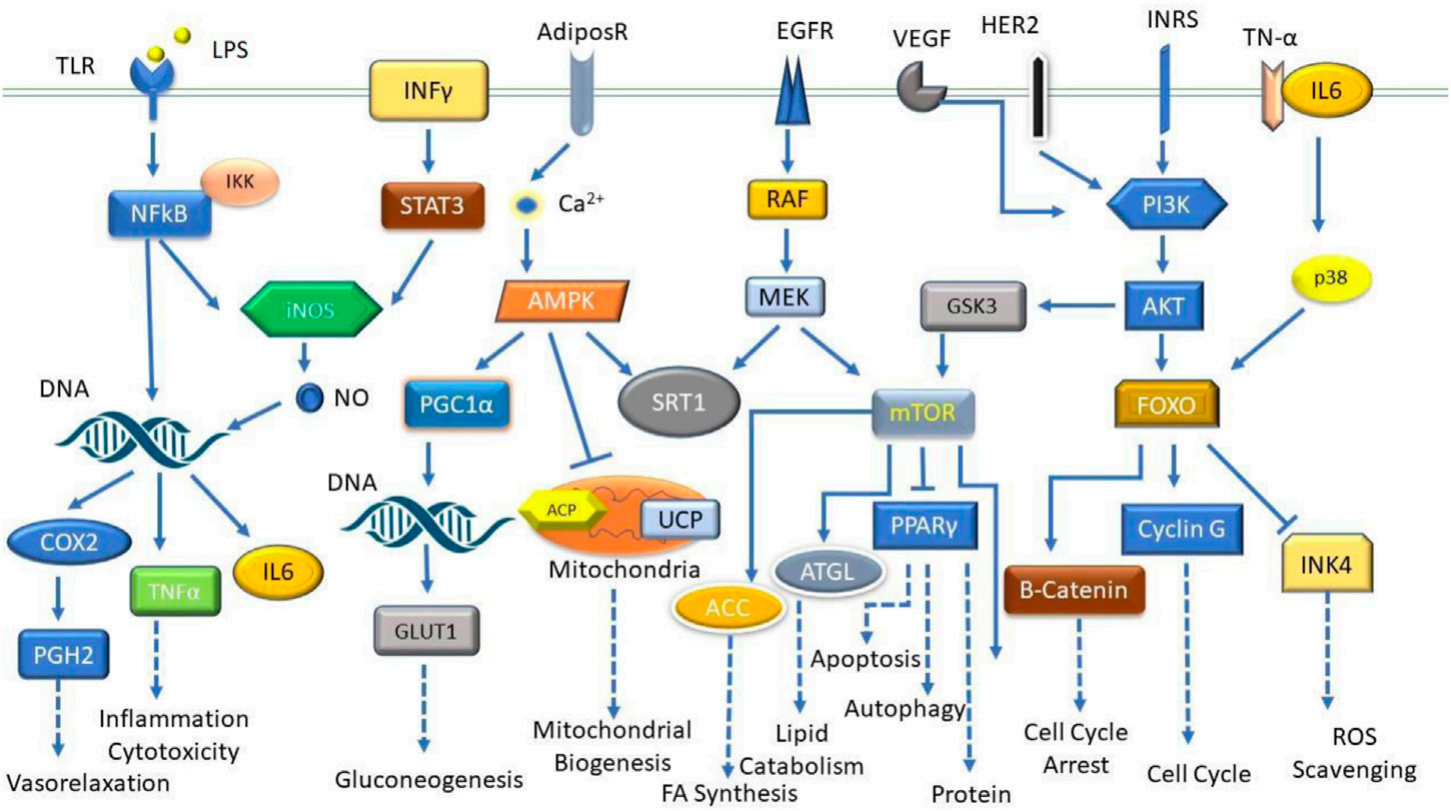
Molecular targets of *Kaempferia galanga, Kaempferia parviflora and Kaempferia rotunda*.

Figure 5 lists the molecular targets and actions of KG, KP and KR.

## 7 Biotechnological intervention

Biotechnological interventions in the *Kaempferia* genus mainly comprise tissue culture, *in vitro* cell studies, and genetic diversity analysis using molecular markers. Apart from this, molecular techniques like transcriptome sequencing and digital gene expression (DGE) have also been applied to *Kaempferia* spp. however, to date, proteome and metabolome-based studies are not done in this genus.

### 7.1 Tissue culture and *in vitro* rhizome induction

Tissue culture is an indispensable technique for the rapid and pathogen-free propagation of plants. It is a very well-explored tool for crop improvement and sustainable growth. The microrhizomes (produced *in vitro*) can be efficiently used by farmers. Rhizome induced *in vitro* propagation yields plantlets that are easy to transport with minimum injury.

KP’s rhizomes can be used to multiply the plant (Prathanturarug et al.,2007; Labrooy et al., 2016). A mature rhizome requires a year to propagate (Labrooy et al., 2020). Microrhizome synthesis, *in vitro* plant regeneration, and KP cell suspension-based culture have all been documented (Nazreena et al., 2014; Labrooy et al., 2016) (Zuraida et al., 2015; Labrooy et al., 2016). The rhizome of KP was used for developing an *in vitro* plant regeneration technique in MS media. Plantlets of KP were adapted in a growing chamber for 2 weeks, and 98 per cent of them survived. Additionally, Park *et al.* claimed that growth was achieved when the surface of rhizome buds was sterilized with silver nanostructures rather than sodium hypochlorite (Park et al., 2021).

Studies have used several plant growth enhancers, including benzyladenine, indoleacetic acid, indolebutyric acid, naphthalene acetic acid, and adenine sulphates (Ads), for multiple shoot induction in KG*,* Parida et al., 2010 discovered that adding BA (1 mg/L) and IAA (0.5 mg/L) to MS led to a significant increase in the number of shoots. They also optimized the media for high production of leaf biomass which contained 1 mg/L BA and 0.5 mg/L IAA. Further, the results of the RAPD analysis showed that the micro propagated plants displayed genetic stability (Parida et al., 2010). In the year 2011, Mohanty *et al.* used MS agar medium to proliferate KG *in vitro* (micro) by inoculating explants and developed a method for fast micropropagation and *in vitro* leaf biomass propagation. Vidya et al. (2022) observed the strongest microrhizome induction with the highest dose of used AgNPs (25 mg/L) in MS medium with 0.1 mg/L TDZ (thidiazuron) and 2.0 mg/L NAA.

Another study revealed that KR pseudostem explants developed more shoots when they were grown in liquid MS media in comparison with the MS agar. Activated charcoal (AC) had a positive effect on the plantlet height while a negative impact on the number of shoots produced. On the other hand, there was no difference in the quantity and size of leaves, the number of roots, or the length of the roots (Sotthikul and Potihongsa, 2017). The embryogenic callus of KR was successfully grown using an MS solid medium that was supplemented with 2.5 mg/L 2,4-D and 0.5 mg/L BAP.

Globoid or torpedo-shaped Somatic embryos were taken from callus culture and enclosed in calcium alginate beads before being transplanted successfully into the field, where they were established with a 50% success rate (Mustafaanand 2014). The commercial application of the approach, which requires the mass production of true-to-type plants of a certain genotype on a massive scale, depends on the genetic stability of tissue-grown plants (Mohanty et al., 2011). A small number of reports on tissue culture studies in KG that have been published so far (Shirin et al., 2000; Rahman et al., 2005) do not mention any work on the *in vitro* production of commercially useful extractable leaf biomass.

### 7.2 Molecular biology studies

Molecular studies conducted on the genus *Kaempferia* have emphasized species identification using molecular markers, phylogenetic analysis and genetic variations.

In 2015, Preetha et al. (2015) compared the variations in the RAPD banding pattern of the cryopreserved samples, somatic embryo derived samples and control. They noticed some variation in the somatic embryo-derived samples due to the callus phase whereas no variation was seen in the cryopreserved materials. Rajasekharan et al. (2017) collected four accessions from the southern part of India and demonstrated genetic differentiation and genetic diversity utilising inter simple sequence repeat (ISSR) markers. Overall, significant genetic differentiation and non-significant genetic diversity were obtained among the four populations of KG. These results were further useful in establishing conservation policy. Devi et al. (2015) reported distinct diversity in the eight cultivars of KG from Manipur, North-East India using ISSR markers and cluster analysis by RAPD. In another study Subositi et al. (2020) employed ISSR markers for studying genetic diversity in 12 different accessions of KG. High-level genetic diversity was seen in KG with a genetic similarity index ranging from 49.6% to 93.3%. In order to use *Kaempferia* on a sustainable basis*,* a broader phytochemical and cytogenetic investigation is required. Bhadra et al. (2020) explored four species of *Kaempferia* (*K. rotunda* L., *K. galanga* L., *K. elegans* (Wall.) Baker and *K. angustifolia* Roscoe). A total of eight accessions were used for ISSR and RAPD based analysis and chemotypic differences in *Kaempferia* were indicated in the results. Researchers have exploited techniques like thin-layer chromatography (TLC) image analysis and TLC-densitometry that could run a quality evaluation of volatile oils of *Kaempferia* spp (Pitakpawasutthi et al., 2018).

In a recent study by Joothamongkhon et al. (2022), chemical markers responsible for the green-leaf types and red-leaf types of KP were explored. They collected the samples of KP from 39 different locations in Thailand, assessed the genetic diversity and constructed a population structure. Their study introduced another angle to discriminate the two types of KP on the bases of chemical profiling. In 2014 first report on *de novo* transcriptome data of *K. pandurata* Roxb. Was published. The data provided the pathway of panduratin A production and regulation of involved genes (Md-Mustafa et al., 2014). For a better understanding of the evolution of Zingiberaceae species, chloroplast genomes of *K. galanga* L. and *K. elegans* (Wall.) Baker were sequenced in 2019. The results helped in presenting a picture of chloroplast DNA evolution within *Kaempferia* spp (Li et al., 2019)*.*

## 8 Industrial uses, importance and prospects

The rhizome extracts of *K. rotunda* L.*, K. parviflora* Wall. Ex Baker*, K. galanga* L.*, K. pulchra* Ridl.*, K. elegans* (Wall.) Baker*, K. angustifolia* Roscoe*,* and *K. marginata* Carey ex Roscoe have been tested for their UV protection and antioxidant properties. A chalcone discovered in *K. elegans* (Wall.) Baker called flavokawain B protects against UVA and UVB radiation and has been found to be more efficient than commercial sunscreens (Panyakaew et al., 2021). Gold nanoparticles (AuNPs) based on KP were created (50 ml of Millipore-MilliQ distilled water was used to make a stock solution of chloroauric acid (HAuCl4) (14.6 mM) using 261 mg of Au (gold) precursor and diluted to 0.1 mM concentration.1.0 ml rhizome extract of KP mixed with 4 ml of 0.1 mM chloroauric acid was mixed at 200 rpm for 30 min. UV-vis spectra were further used to confirm the formation and stability of BG AuNPs.), and they were proved to be the ideal replacement for artificial nanomaterials. Along with anti-inflammatory, antioxidant, anticancer, and antibacterial effects, they have demonstrated biological and environmental applications. Additionally, it was discovered that the AuNPs were a potent catalyst for the breakdown of methyl orange using sodium borohydride (Varghese et al., 2021).

*Kaempferia* spp. was used to extract the lipophilic metabolites, carotenoids, fatty acids, vitamin K1, phytosterols, and tocopherols. All these have been utilized as antioxidants in the nutraceutical, culinary, and cosmetic sectors.

The lipophilic antioxidant profile of *in vitro* and *ex-vitro* cultivated KP plants was investigated because the leaves of KP represented a substantial byproduct of their manufacturing. With repeated reaction monitoring and liquid chromatography-mass spectrometry, many lipophilic substances were measured. Compared to leaves produced *in vitro*, *ex-vitro* leaves contained higher concentrations of the total carotenoids, lutein, -tocopherol, -carotene, neoxanthin, -carotene, violaxanthin, -linolenic acid, palmitic acid, oleic acid, and palmitoleic acid. These results suggest that *ex-vitro* grown KP leaves can be an advantageous natural source for extracting necessary lipophilic antioxidants (Song K. et al., 2021). The demand for essential oils of KG has grown both nationally and internationally therefore it is necessary to find a high yielding good quality chemovar. Singh S. et al. (2022) claimed that the chemovars Kg16 and Kg14 produced the desirable constituents and best oil in terms of quality and quantity. Worldwide acceptance of KG’s antibacterial function as a natural preservative for meals based on poultry is well known. The presence of acetic acid in KG may help explain its antibacterial properties (Song L. et al., 2021). Lectin polypeptides mediate KR’s anti-cancer effects (Rashel Kabir et al., 2011; Islam et al., 2019). When administered to mice in the form of silver/silver chloride nanoparticles, it also inhibits the formation of tumors (Kabir et al., 2020).

It may be possible to improve treatment effectiveness for the goal and desired pharmacokinetic profile by doing extensive research on nanoparticle drug delivery techniques in clinical practice. Furthermore, KG has the potential to evolve into a phytopharmaceutical against oral mucosal ulcers in people as revealed in an investigation after receiving clinical approval. It is mandatory to run clinical investigations prior to certifying the usage of any such medical formulation (Wahyuni et al., 2022).

## 9 Conservation strategies

Due to its numerous uses in the production of drugs, cosmetics, pharmaceuticals, and ayurvedic medicines, the genus *Kaempferia* is being harvested indiscriminately as raw material. Due to the depletion of this natural resource, they are now facing more challenges (Labrooy et al., 2016). Despite being one of the most important medicinal plants in tropical Asia, conservation methods have not been well devised for these plants. Many *Kaempferia* species are endangered or unusual, so, it is very important to strategize their preservation for sustainable use (Ravikumar et al., 2000).

The yield is poor in traditional cultivation methods that include vegetative proliferation through rhizomes (Preetha et al., 2016). Therefore, cost-effective solutions are required for the mass production of these plants. Several plant species with high medicinal values have been successfully preserved using *in vitro* procedures (Jacob et al., 2004; Piovan et al., 2010; Phulwaria et al., 2012; Cheruvathur et al., 2013). However, KG is the only thrust species for which conservation efforts have been done so far. For *in vitro* propagation of KG, techniques like organogenesis and multiple shoot induction using various plant tissues as explants, including rhizome (Vincent et al., 1991; Chithra et al., 2005; Kalpana et al., 2009), shoot bud (Bhattacharya et al., 2013), and leaf base (Lakshmi et al., 2003; Rahman et al., 2005; Preetha et al., 2008) have come handy.

KG shoot meristem cryopreservation using an innovative technique was demonstrated by Preetha et al. (2021). The usefulness and viability of cryopreservation in long-term conservation measures for KG were demonstrated by them.

## 10 Conclusion

*Kaempferia* species are among the oldest and most widely used botanical drugs in tropical Asian traditional medicine. The most recent studies and information on their distribution, relevance, industrial uses, phytochemistry, ethnopharmacology, tissue culture, as well as their cultivation and preservation, are compiled in this review. It also describes their numerous and varied biological activities. The development of analytical methods has led to a major increase in our understanding of how *Kaempferia* spp. Act biologically and in the discovery of several new compounds. Our analysis revealed that although widely used in traditional medicines, many *Kaempferia* species are not scientifically studied. Most of the biological activity evaluation studies are performed using *in vitro* studies and animal based and clinical studies are very limited. Similarly, bioassay guided isolation studies are performed to isolate and identify the active constituents in these plants. Quantification of bioactive compounds and development of standardization protocolas are necessary to expand the commercial use of these species in future.

New methods have also improved the utilization of these species in the manufacturing of commercial sunscreen lotion, nanoparticle creation, among other industrial applications. The usage of medicines made from plants such as *Keampferia* species holds the potential to raise economic standing and improve the standard of living in developing nations. However, the wild population of such medicinally important botanical drugs in the environment might be threatened because of their overexploitation. Thus, conservation strategies need to be formulated for their sustainable use. The quantitative ratio of bioactive metabolite production and biological activities is greatly influenced various factors such as genetic, environmental, processing, *etc.* Therefore, it is also important to study these parameter in detail. This calls for a thorough assessment of genetic diversity and plant physiology. In order to hasten the proper use and conservation of the genus *Kaempferia*, it is also essential to pay special attention to international regulations. The issue of access and benefit-sharing (ABS) is discussed on a worldwide scale for the objective distribution of advantages among countries with high biodiversity and user enterprises. Input and focus from scientists should grow in order to preserve and use *Kaempferia* spp. in the best possible ways.
